# Internal dosimetry study of [^82^Rb]Cl using a long axial field-of-view PET/CT

**DOI:** 10.1007/s00259-024-06660-7

**Published:** 2024-02-26

**Authors:** Lorenzo Mercolli, Carola Bregenzer, Markus Diemling, Clemens Mingels, Axel Rominger, Hasan Sari, Sigrid Seibel, Antti Sohlberg, Marco Viscione, Federico Caobelli

**Affiliations:** 1 Department of Nuclear Medicine, Inselspital, Bern University Hospital, University of Bern, Bern, Switzerland; 2 Hermes Medical Solutions, Stockholm, Sweden; 3 Advanced Clinical Imaging Technology, Siemens Healthcare AG, Lausanne, Switzerland; 4 Department of Clinical Physiology and Nuclear Medicine, Päijät-Häme Central Hospital, Lahti, Finland

**Keywords:** Dosimetry, Long axial field-of-view PET/CT, Myocardial perfusion imaging, Rubidium-82

## Abstract

**Purpose:**

Long axial field-of-view (LAFOV) positron emission tomography (PET) systems allow to image all major organs with one bed position, which is particularly useful for acquiring whole-body dynamic data using short-lived radioisotopes like ^82^Rb.

**Methods:**

We determined the absorbed dose in target organs of three subjects (29, 40, and 57 years old) using two different methods, i.e., MIRD and voxel dosimetry. The subjects were injected with 407.0 to 419.61 MBq of [^82^Rb]Cl and were scanned dynamically for 7 min with a LAFOV PET/CT scanner.

**Results:**

Using the MIRD formalism and voxel dosimetry, the absorbed dose ranged from 1.84 to 2.78 μGy/MBq (1.57 to 3.92 μGy/MBq for voxel dosimetry) for the heart wall, 2.76 to 5.73 μGy/MBq (3.22 to 5.37 μGy/MBq for voxel dosimetry) for the kidneys, and 0.94 to 1.88 μGy/MBq (0.98 to 1.92 μGy/MBq for voxel dosimetry) for the lungs. The total body effective dose lied between 0.50 and 0.76 μSv/MBq.

**Conclusion:**

Our study suggests that the radiation dose associated with [^82^Rb]Cl PET/CT can be assessed by means of dynamic LAFOV PET and that it is lower compared to literature values.

**Supplementary Information:**

The online version contains supplementary material available at 10.1007/s00259-024-06660-7.

## Introduction

Due to its cost-effectiveness, [^82^Rb]Cl is increasingly used in positron emission tomography (PET) for myocardial perfusion imaging (MPI) [1, 2]. With a broader clinical adoption, it is important to have accurate and reliable estimates for the radiation dose that is delivered to the patient. However, only a few studies investigated dosimetry with [^82^Rb]Cl PET/CT, and wide discrepancies can be seen in the reported values [3–8].

Previous biokinetic and dosimetric studies for [^82^Rb]Cl are plagued by the short half-life of the radiotracer (76 s) and by the use of analogue PET/CT with a standard axial field-of-view (SAFOV). Moreover, while it is theoretically possible to use blood flow as a surrogate quantity to estimate the biokinetics of [^82^Rb]Cl [3, 8], the quantification of the strong model dependence is challenging. Note also that SAFOV PET/CT protocols require multiple bed positions and therefore longer scan times to image all relevant organs [4, 5, 7] and a whole-body dynamic acquisition is not feasible [9]. The introduction of long axial field-of-view (LAFOV) PET/CT systems in clinical routine [10, 11] enables whole-body dynamic imaging and kinetic modelling with unprecedented accuracy [12, 13]. This allows in turn for an accurate estimate of the absorbed radiation dose.

The aim of this study is to increase the accuracy of the dose estimates for [^82^Rb]Cl imaging, which were to date hampered by the use of surrogate quantities (blood flow) or large injected activities (to compensate for long scan times). To that end, we estimated the absorbed doses in healthy volunteers, by acquiring dynamic [^82^Rb]Cl PET images on a LAFOV PET/CT scanner.

## Materials and methods

### Subjects

**Fig. 1:**
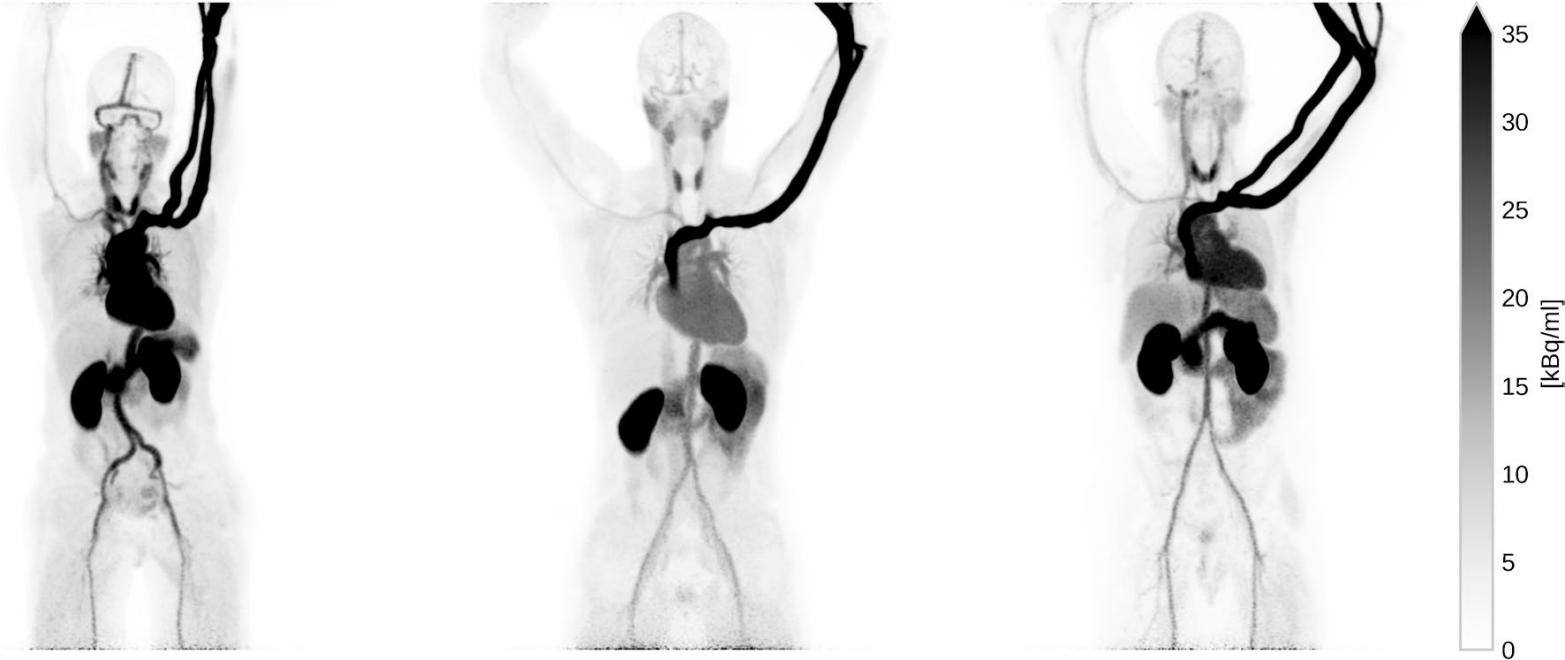
Maximum intensity projection (MIP) of the three subjects at rest. The images depict the full scan integration

Three healthy volunteers were scanned first at rest and immediately after under stress conditions. Pregnancy was excluded at the time of acquisition and none had diffuse atherosclerosis or coronary artery disease (CAD). The pharmacological stress was induced with 400 mcg of Regadenoson. The injected [^82^Rb]Cl activity ranged from 407.0 to 419.61 MBq. The subjects’ characteristics and the administered activities of [^82^Rb]Cl are displayed in Table 1. [^82^Rb]Cl was produced with CardioGen-82 radionuclide generator and infusion system (Bracco Imaging S.p.A, Milan, Italy). The radiotracer was automatically infused in an antecubital vein of the left arm over 20 s. In Fig. 1, the maximum intensity projections (MIP) images of the 3 subjects are shown.

**Table 1:** Personal details of the three subjects and administered activity for the rest and stress examinations

Subject	Gender	Age [y]	Weight [kg]	Height [m]	Activity rest [MBq]	Activity stress [MBq]
P1	F	57	57	1.65	407.00	407.03
P2	M	29	88	1.96	404.59	409.19
P3	M	40	84	1.72	398.43	401.73

### Imaging protocol

Images were acquired on a LAFOV PET/CT scanner (Biograph Vision Quadra, Siemens Healthineers, Knoxville, TN, USA) [14]. The scans started simultaneously with the administration of [^82^Rb]Cl and electrocardiogram-ungated images were acquired in list mode over 7 min both at rest and under stress.

The images were reconstructed using a dedicated image reconstruction prototype (e7-tools, Siemens Healthineers) to a 440 × 440 matrix, with 3-mm slice thickness, 4 iterations, 5 subsets, time-of-flight (TOF), point-spread-function (PSF) recovery, and a 2-mm full width at half maximum Gaussian filter. Seven time points were reconstructed for each scan, i.e., integrating the accumulated counts between 0 and 30, 30 and 60, 60 and 90, 90 and 120, 120 and 180, 180 and 270, and 270 and 420 s, respectively. Each image was reconstructed using a proprietary ordered-subset expectation maximum (OSEM) iterative algorithm. The images were corrected for attenuation and scatter based on a low-dose CT (dose-length product 27.4 to 41.6 mGy cm).

### Dosimetry

We retrospectively determined the time-activity curves (TACs) from the LAFOV PET images of each subject. To this end, 11 source organs (adrenals, gallbladder, stomach, heart wall/ventricular cavities, kidneys, liver, lung, spleen, thyroid, urinary bladder, uterus) were segmented using the corresponding CT images and applied to all time points in the PET images. The organ segmentation was performed using the artificial intelligence–based TotalSegmentator tool [15] and an experienced nuclear medicine physician verified the automatic segmentation as needed (the segmentation is shown in Supplemental Fig. 1). For the TACs, the mean intensity value has been decay corrected for the decay during each frame and to the beginning of the scan.

To compute the dose based on the MIRD formalism [16], we applied a double exponential fitting function to the seven time points of the TACs and removed the decay correction to the injected activity (hence only the decay within a time frame is corrected). The fitted function was integrated over time in order to obtain the time-integrated activity (TIA) for each source organ. We used the subjects’ TIA as input for Olinda/EXM version 2.2.3 (Hermes Medical Solutions, Stockholm, Sweden) to compute the organ doses for the ICRP adult male and female reference phantoms [17].

As a second method, we used a prototype voxel dosimetry software (Hermes Medical Solutions, Stockholm, Sweden) to compute a fully individual patient dose. The voxel-wise TIA is computed through a trapezoidal time integration over the seven time points and a mono-exponential decay after the last time point. The decay parameter of the mono-exponential decay is fitted from the last two time points. The same organ segmentation of the PET images is applied to the resulting dose maps to obtain the organ doses.

## Results

**Fig. 2:**
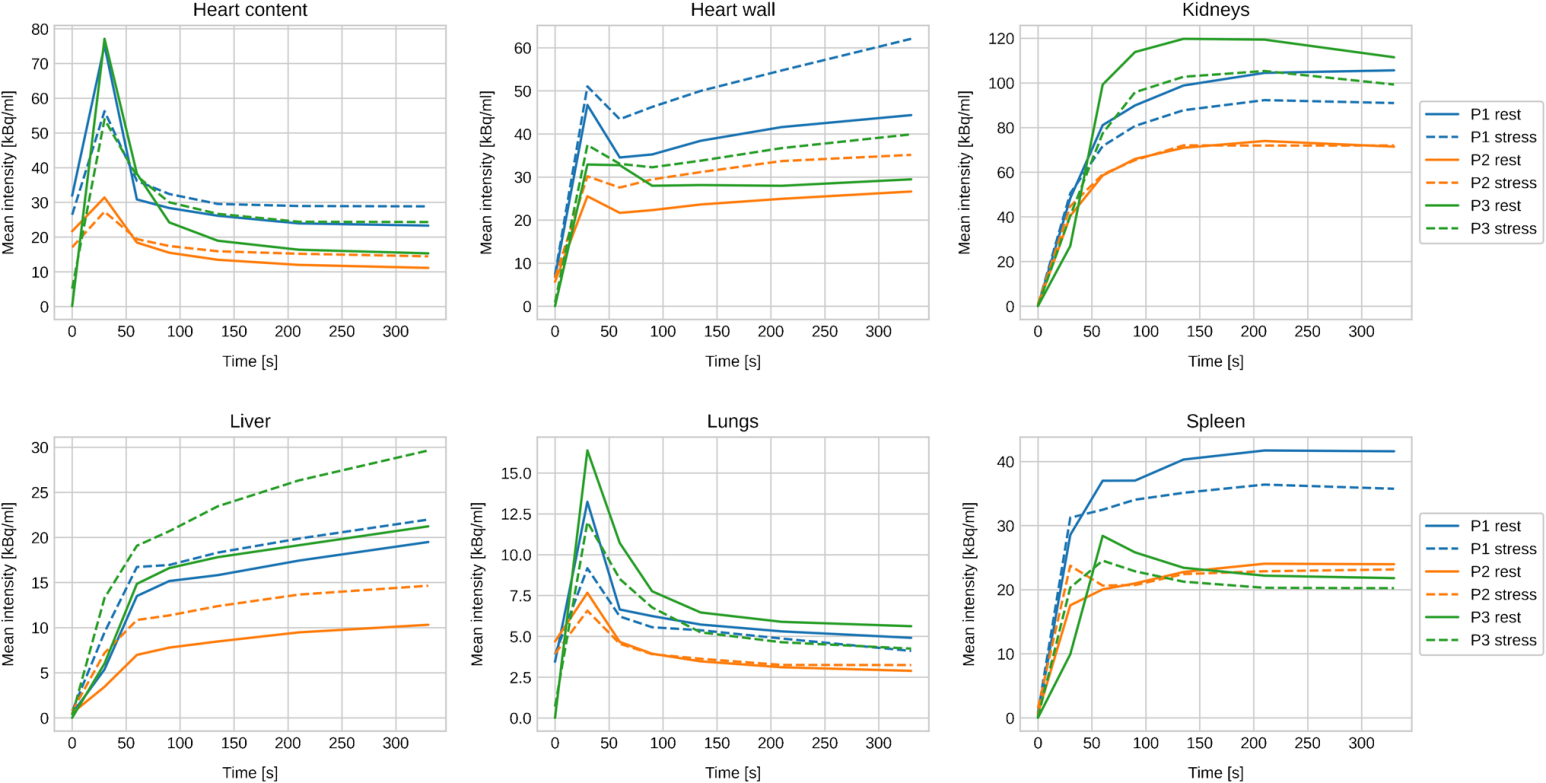
Decay corrected TAC of selected organs

In Fig. 2, we show the TACs of selected organs at rest and under stress. These values were obtained by integrating a double exponential fitting function over time. All organs show a rapid increase within the first minute, which stabilizes after about 2 min. In Table 2, we report the TIA for the three subjects at rest and under stress.

**Table 2:** Normalized TIA in [MBq h/MBq] for the segmented source organs in all three subjects at rest and under stress. As a comparison, we show also the reference TIA from [8]. The total body TIA is the remaining activity that is not taken into account by a segmented organ

Source organ	ICRP 128 [MBq h/MBq]	P1 rest [MBq h/MBq]	P1 stress [MBq h/MBq]	P2 rest [MBq h/MBq]	P2 stress [MBq h/MBq]	P3 rest [MBq h/MBq]	P3 stress [MBq h/MBq]
Adrenals	0.000046	0.000003	0.000003	0.000005	0.000005	0.000008	0.000009
Gallbladder content	NA	0.000005	0.000008	0.000003	0.000004	0.000001	0.000003
Stomach content	NA	0.000278	0.000311	0.000181	0.000226	0.000320	0.000460
Heart content	0.0013	0.001142	0.001131	0.001117	0.001069	0.001116	0.001076
Heart wall	0.00094	0.000291	0.000378	0.000300	0.000383	0.000290	0.000331
Kidneys	0.0033	0.001008	0.000929	0.001373	0.001376	0.002221	0.001929
Liver	0.0018	0.001318	0.001616	0.001141	0.001722	0.001582	0.002164
Lungs	0.0029	0.002360	0.002086	0.002040	0.001892	0.001723	0.001367
Spleen	0.00062	0.000291	0.000270	0.000257	0.000262	0.000416	0.000409
Thyroid	0.000038	0.000034	0.000031	0.000025	0.000027	0.000036	0.000031
Urinary bladder content	0.000044	0.000017	0.000033	0.000035	0.000032	0.000023	0.000028
Total body	NA	0.023489	0.023441	0.023760	0.023239	0.022499	0.022431

**Fig. 3:**
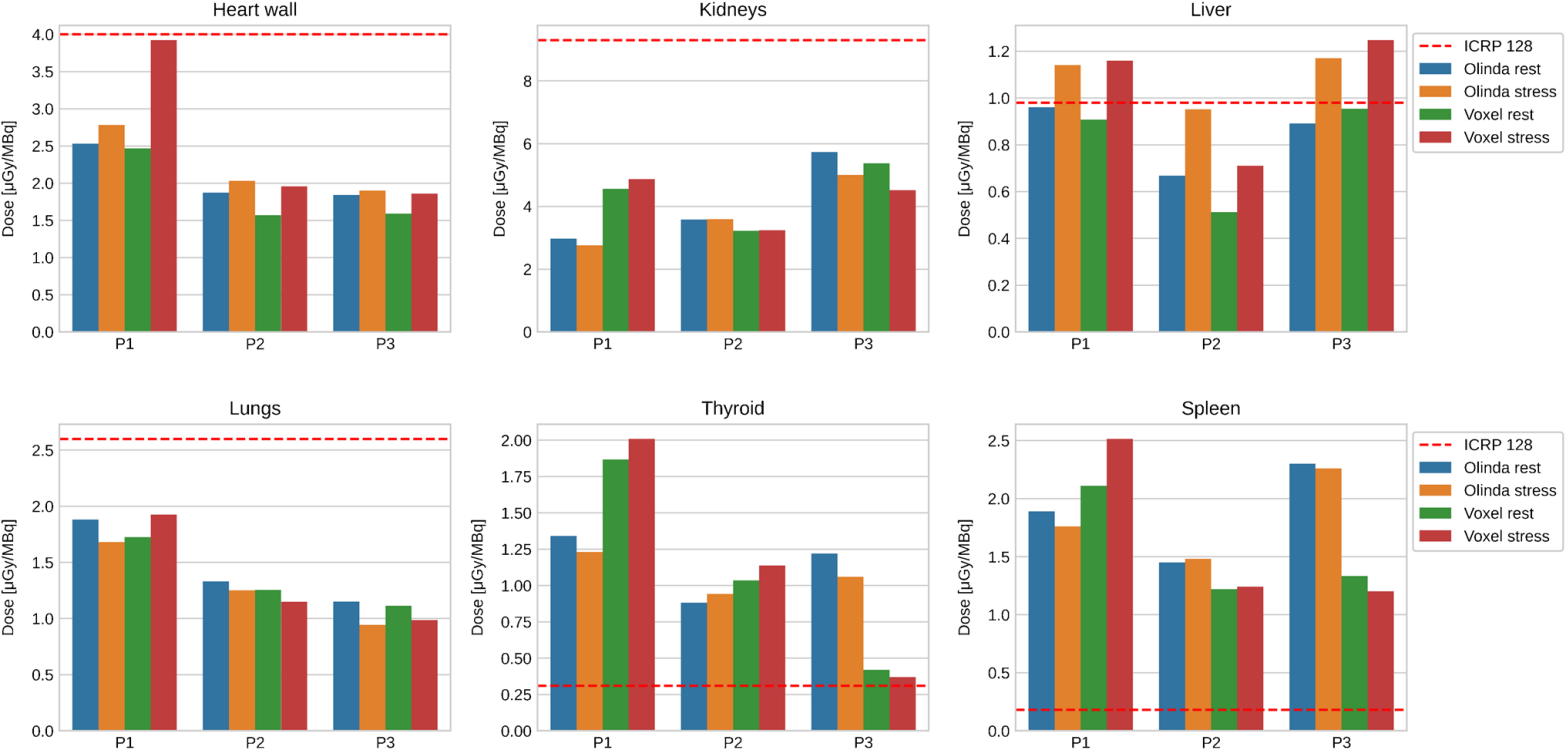
Normalized absorbed dose for selected organs as obtained from Olinda/EXM and voxel dosimetry. The dashed line indicates the corresponding dose from the ICRP

In Table 3, we report the normalized organ doses as obtained from Olinda/EXM. For the most relevant organs, we visualize the absorbed doses in Fig. 3, where we added also the organ doses computed from Hermes’ voxel dosimetry module (see Table 4 for the full voxel dosimetry results).

**Table 3:** Normalized absorbed doses for the target organs from Olinda/EXM in [μGy/MBq]. The ICRP 128 [8] uses only one adult phantom, while Olinda/EXM reports the organ doses for the standard ICRP male and female phantoms separately. Hence, some organs are not applicable depending on the subjects’ gender. In addition, some target organs from Olinda/EXM are not reported in the ICRP 128 [8], like, e.g., the eyes or salivary glands

Target organ	ICRP 128 [μGy/MBq]	P1 rest [μGy/MBq]	P1 stress [μGy/MBq]	P2 rest [μGy/MBq]	P2 stress [μGy/MBq]	P3 rest [μGy/MBq]	P3 stress [μGy/MBq]
Adrenals	2.4	0.758	0.744	0.754	0.786	1.15	1.15
Brain	0.14	0.402	0.401	0.332	0.325	0.315	0.314
Breasts	0.19	0.418	0.417	NA	NA	NA	NA
Esophagus	1.5	0.524	0.528	0.421	0.422	0.411	0.412
Eyes	NA	0.402	0.401	0.332	0.325	0.315	0.314
Gallbladder wall	0.72	0.526	0.557	0.461	0.501	0.466	0.516
Heart wall	4.0	2.53	2.78	1.87	2.03	1.84	1.9
Kidneys	9.3	2.97	2.76	3.58	3.59	5.73	5.0
Left colon	NA	0.491	0.492	0.397	0.395	0.396	0.395
Liver	0.98	0.96	1.14	0.668	0.951	0.891	1.17
Lungs	2.6	1.88	1.68	1.33	1.25	1.15	0.943
Osteogenic cells	NA	0.267	0.266	0.26	0.256	0.251	0.249
Ovaries	0.5	0.45	0.45	NA	NA	NA	NA
Pancreas	2.6	0.525	0.534	0.415	0.423	0.424	0.434
Prostate	NA	NA	NA	0.371	0.364	0.356	0.354
Rectum	NA	0.444	0.444	0.372	0.365	0.355	0.354
Red marrow	0.38	0.357	0.355	0.296	0.293	0.287	0.286
Right colon	NA	0.469	0.47	0.39	0.389	0.383	0.385
Salivary glands	NA	0.415	0.414	0.352	0.345	0.334	0.333
Small intestine wall	2.0	0.451	0.451	0.387	0.383	0.378	0.377
Spleen	0.18	1.89	1.76	1.45	1.48	2.3	2.26
Stomach wall	0.83	1.04	1.1	0.436	0.821	0.982	1.23
Testes	0.26	NA	NA	0.343	0.336	0.325	0.324
Thymus	1.5	0.652	0.638	0.436	0.435	0.417	0.416
Thyroid	0.31	1.34	1.23	0.881	0.941	1.22	1.06
Total body	0.31	0.524	0.524	0.422	0.423	0.423	0.423
Urinary bladder wall	0.18	0.451	0.493	0.43	0.417	0.388	0.397
Uterus	1.0	0.446	0.446	NA	NA	NA	NA

**Table 4:** Normalized organ doses from Hermes’ voxel dosimetry module

Target organ	P1 rest [μGy/MBq]	P1 stress [μGy/MBq]	P2 rest [μGy/MBq]	P2 stress [μGy/MBq]	P3 rest [μGy/MBq]	P3 stress [μGy/MBq]
Adrenals	1.49	1.98	0.989	1.03	1.02	1.05
Gallbladder content	0.574	0.860	0.350	0.412	0.555	0.995
Stomach content	1.38	1.84	0.652	0.776	0.742	0.965
Heart content	2.74	3.08	1.67	1.57	1.37	1.80
Heart wall	2.47	3.92	1.57	1.96	1.59	1.86
Kidneys	4.56	4.87	3.22	3.24	5.37	4.52
Liver	0.907	1.16	0.512	0.710	0.954	1.25
Lungs	1.72	1.92	1.25	1.15	1.11	0.985
Spleen	2.11	2.51	1.22	1.24	1.33	1.20
Thyroid	1.87	2.01	1.03	1.14	0.418	0.370
Urinary bladder content	0.269	0.535	0.111	0.105	0.280	0.306

The total body effective dose is reported in Table 5 and lies between 0.50 and 0.76 μSv/MBq. As expected from the TIA in Table 2, the effective doses for the three subjects are consistently lower than the reference value from the ICRP 128 publication (also depicted in Supplemental Fig. 2) [8].

**Table 5:** Normalized total body effective dose in [μSv/MBq] for the 3 subjects

	ICRP 128μSv/MBq]	P1 rest [μSv/MBq]	P1 stress [μSv/MBq]	P2 rest [μSv/MBq]	P2 stress [μSv/MBq]	P3 rest [μSv/MBq]	P3 stress [μSv/MBq]
Effective dose	1.1	0.757	0.745	0.502	0.553	0.593	0.597

## Discussion

The unique possibility to image all main organs of a patient with a single bed position allows [^82^Rb]Cl LAFOV PET/CT to go beyond the standard paradigms of cardiovascular imaging in patients with suspected or known coronary artery disease (CAD) [9, 18, 19]. Due to the significant increase in sensitivity compared to SAFOV systems, the administered activity can be kept to a minimum [10, 18] and this advantage reflects our choice to administer activities below the to-date recommended range (740 to 1480 MBq) [2]. Hence, the injected activities in our study are significantly lower than those administered in the previous reports [4, 5, 7]. This is also consistent with the manufacturer’s recommendation, suggesting that activities above 555 MBq [^82^Rb]Cl would likely saturate the scanner’s data acquisition.

We found that almost all TIA are smaller than reported in ICRP publication 128 [8]. This is in line with the conservative nature of the ICRP values and they do not represent individual estimates for a single patient. Furthermore, the strong model dependence of [8] conceivably drives the TIA to higher values. Furthermore, our TIA is slightly lower than reported in [4, 7]. While it should be noted that the TAC and TIA vary among the three subjects, the same issue also pertains to the abovementioned reports, wherein the standard deviation across patients in the cohort was quite large.

Consistent with previous studies, we found differences in the TACs between rest and stress acquisitions. Specifically, while some organs show an increased uptake on stress (e.g., heart, liver), others present with higher TACs on rest (e.g., kidneys, spleen). Comparing the rest and stress TIA, our results are consistent with the reports from Senthamizhchelvan et al. and Mattssonn et al. [5, 7], wherein similar differences were seen.

For some target organs, the normalized dose obtained from Olinda/EXM showed an excess compared to the ICRP 128 values (see Table 3 and Fig. 3). Given that TIA in our study is consistently lower than in [8], the most conceivable explanation lies in the differences in human phantoms (and therefore in the S-values) that underlie the ICRP 128 and the Olinda/EXM dose calculation. Furthermore, the normalized total body effective doses shown in Table 2 are smaller than the reports of [7, 8]. Interestingly, [4] report a total body effective dose, which is even slightly higher than [8] and almost double compared to our results. The contention may relate to either the rather large systematic uncertainties in the dose estimation in general or to a possible overestimation of the TIA in [4] due to their fitting procedure that uses only three time points.

On average, the difference between rest and stress in the absorbed dose is marginally lower for the three subjects. For the gallbladder wall, heart wall (only subjects P1 and P2), kidneys (only subjects P1 and P3), liver, lungs, spleen (only subject P1), thyroid and urinary bladder (only subject P1), the relative difference between rest and stress dose exceeds 5%. The resulting total body effective dose reflects the results from the organ doses, i.e., the relative differences are 1.6, − 9.2, and − 0.7% for the subjects P1, P2, and P3, respectively. Given the small difference, a pharmacological-induced stress is unlikely to affect patient’s dose, which is in line with the ICRP’s assumption to disregard the effects of physiological rest and stress on absorbed dose to the patient.

The organ doses from the voxel dosimetry show an overall consistency with the doses from Olinda/EXM. We attribute the deviations seen in Fig. 3 to intrasubject variabilities and the uncertainties in the TIA determination at the voxel as well as at the organ level. This is in line withs previous comparisons between Olinda/EXM and Hermes’ voxel dosimetry [20, 21], albeit in the context of PET tracer rather than therapeutic applications.

The main limitation of our study is the limited number of subjects, due to the current unavailability of cardiac gating for the LAFOF-PET scanner, which forbids the use for clinical routine. Despite the small patients’ cohort, our results provide a strong rationale to further investigate the role of LAFOV PET in dosimetry studies with [^82^Rb]. Moreover, our work suggests that a reassessment Cl dosimetry should be pursued, taking advantage of the possibility to perform “whole-body” dynamic imaging [9]. Second, we did not correct our data for partial volume effect (PVE), and it may be argued that a model of biodistribution may suffer from PVE depending on how it was derived. Indeed, a more pronounced difference should be seen in the voxel dosimetry results, given the fact that the dose is inversely proportional to the volume. However, TIA of the segmented organs should not be significantly affected by PVE, since it is derived from the mean voxel values of the segmented organs. Considering mean voxel values, TACs in our study show consistency across patients. Hence, it is conceivable that the impact of PVE is not relevant.

Summarizing, our study shows that LAFOV PET/CT can be used to estimate absorbed dose from [^82^Rb]Cl imaging. Our results hint towards lower values of the normalized absorbed dose compared to the literature, while the use of LAFOV PET/CT also enables the use of very low-activity protocols. This gives more reliance in suggesting [^82^Rb]Cl PET/CT in clinical practice with acceptable radiation exposure. Similarly, to previous reports, TACs and TIA varied to a certain extent among the three subjects; hence, inter-subject discrepancies should be considered.

## Conclusion(s)

[^82^Rb]Cl internal dosimetry plays a key role in the overall assessment and optimization of MPI. Our study provides absorbed dose estimates at rest and under pharmacological stress, showing lower values than currently reported in the literature. Hence, it should be of less concern to use [^82^Rb]Cl PET in the assessment of patients with suspected or known coronary artery disease. In addition, the increased sensitivity of LAFOV PET/CT systems allows for a significant reduction of the administered activity, thus allowing a further reduction of the absorbed dose compared to standard SAFOV PET/CT scanners.

### Supplementary Information

Below is the link to the electronic supplementary material.

Supplementary file1 (DOCX 1019 KB)

## Data Availability

The datasets generated during and/or analyzed during the current study are available from the corresponding author on reasonable request.
