# Supplementary material for: Internal dosimetry study of [82Rb]Cl using a long axial field-of-view PET/CT
Source: Eur J Nucl Med Mol Imaging. 2024 Feb 26;51(7):1869–75. doi: 10.1007/s00259-024-06660-7 (PMC11139737; doi:10.1007/s00259-024-06660-7)
Supplement: Supplementary file 1 — Supplementary file1 (DOCX 1019 KB) [file 259_2024_6660_MOESM1_ESM.docx]

# Supplementary material

## Figures


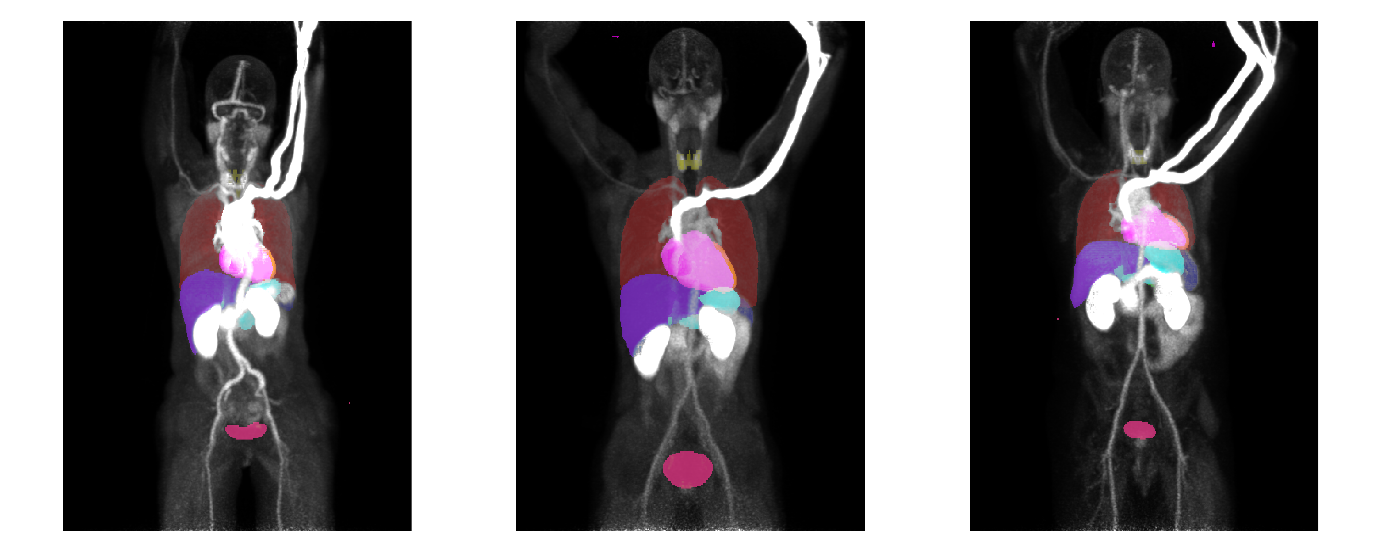


Supplemental Figure 1: Maximum intensity projection (MIP) of the full scan reconstructions with the organ segmentation.


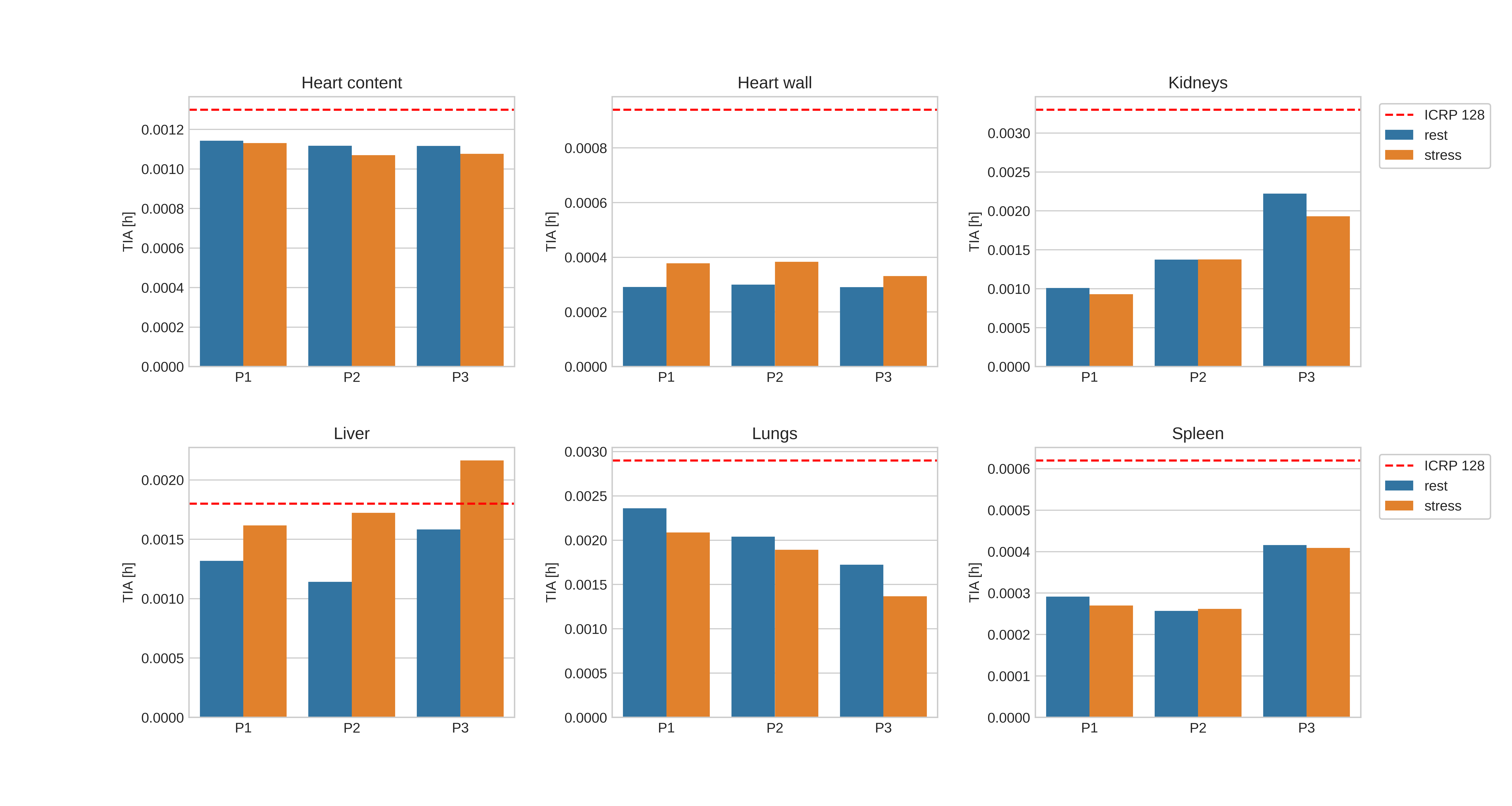


Supplemental Figure 2: Visualization of the normalized time-integrated-activity (TIA) in [MBq h/MBq] for selected organs. The dashed line represents the literature value from ICRP 128 [[8](#ref-icrp128)].
